# On Local Anæsthesia and Electricity

**Published:** 1858-11

**Authors:** Benjamin W. Richardson

**Affiliations:** Physician to the Royal Infirmary for Diseases of the chest, and Lecturer on Physiology at the Grosvenor-place School of Medicine


*On Local Anæsthesia and Electricity* By Benjamin W. Richardson, M. D., L. R. C. P. Physician to the Royal Infirmary for Diseases of the chest, and Lecturer on Physiology at the Grosvenor-place School of Medicine.

In this short communication, I beg to relate the history of certain experimental inquiries having reference to the application of the electric current for the production of anæsthesia. I would premise that it was no intention of mine to publish at this moment. The paper is one long held in reserve for the purpose, after further research, of being sent to one of the learned Societies. But events often check dispositions, and do so now. In the *times* of to-day (Saturday) there is a letter from Mr Sanpe, of Chester, on the application of the electric current for preventing pain in dental operations; and as the subject of this letter, now widely circulated about the world, bears directly on certain points which have received from me much attention, I feel it a duty to hesitate no longer, but at once submit such labours as I have performed to the notice of the Profession.

It is a matter of regret that the results to be given are initiative only, and up to this moment negative in character; the circumstances which have led to their publication must be the excuse for all deficiencies.

My attention was first drawn to the subject in hand in the year 1853, in the following manner;—

I was then engaged in investigating by experiment the influence of electricity on the blood in the living animal body. In one of these experiments a small dog was subjected to an electrical shock, resulting from the discharge of a battery of seventy-two Leyden jars.—Wire chains ready for connexion with the battery were placed one around the throat of the animal, meeting over the upper part of the head, the other around the lower part of the body at the loins. The whole charge was at once passed through the body. The animal fell without a struggle, and lay before me to external appearance, dead.—There was no respiration for several seconds, but the heart continued beating. A little later, and there was a feeble respiratory gasp. I pricked the nose of the animal with the point of a scalpel, and blood issued, but no indication of sensibility on the part of the animal followed. A minute more, and I had laid bare about an inch of the right jugular vein. I tapped the vein, drew off a few drachms of blood for after observation, passed a ligature round the vessel above the opening, brought the edges of the flesh-wound neatly together and secured them by suture. By the time I had done, the signs of reanimation were well marked, but the operation had been performed without the slightest evidence of suffering. For a little time the respiration was short and irregular, but in a few minutes the animal rose slowly, looked about him, as if wondering where he had been, and recovered without a bad symptom.

This was probably the first instance in which any operation was performed without pain, by means of electricity. This result of the experiment was purely accidental. The experiment was originally intended for a different object altogether; but accustomed to operate on narcotized animals, the new fact of the perfect production of insensibility by electricity changed the intention of the experiment entirely in my mind. The idea of producing general anæthesia for the purpose of an operation, by a repetition of this one electrical experiment, was of necessity out of the argument, for it were impossible so to adjust a shock as to produce a sufficient degree of general insensibility for an operation, without the hazard of destroying life altogether. The fact of the production of insensibility was, however, striking; and this fact at once suggested to me that what could be done to the whole body might possibly be done to a part.

To carry out the inquiry which had thus been presented, I tried the effect of passing electrical shocks of varyiug intensities through the limbs of animals. The shocks were severely felt in these cases; but I could never detect that at any instant after the shock the sensibility of the parts through which it had passed was at all destroyed. There was often some temporary twitching of the mnscles of the limb operated on, but the merest attempt to produce pain succeeded.

I next tried vaaious experiments on myself. I charged twenty Leyden jars, and discharged them, either in combination, or one after the other in rapid succession, through one of my fingers. The shocks were painful to bear, and when many were given, the last was felt as severely as the first; but afterwards the finger was as sensitive to a prick from the point of a lancet as it had been previously.

I tried the local effect of the continuous current for long periods, but with as little success.

I passed the electro-magnetic current through one finger for long periods, modifying the intensity of the shocks; sometimes submitting the part for periods of an hour, or more, to a gentle current; at other times increasing the force till the pain produced was scarcely endurable. On January 3, and on August 8, of the present year, I kept a finger for two hours thus exposed; but in these, as in all other cases, without the slightest effect in removing sensibility. While the finger was being subjected to the current, I tested its sensibility by pricking it with a lancet or needle. This test is, however, unnecessary, for so long as the part operated on is sensible to shock, it is sensible to a cut or puncture. An animal deeply narcotized with chloroform is as little sensitive to eleetrical shocks as it is to the knife.

In its local application, indeed, it seems to me that the electric current restores rather than destroys sensibility. One experiment will explain this. Let two fingers be placed in a freezing mixture and held there until the external surface is so benumbed, that the prick of a needle is not felt. Let them then be removed, and let pass through one the current from the electro-magnetic battery. In the finger thus operated upon, it will be found not simply that the sensibility will come back more quickly, but so much the more quickly as to lead to the unpleasant and painful reaction called vulgarly "hotache." The current acts like warmth in this respect.

In one experiment the effects produced were very peculiar, and deserve special note. I placed the first and second fingers of the left hand in a mixture of ice and salt till they were entirely insensible to puncture. I then removed them from the mixture, and, after well drying them, I placed one wire from the electro-magnetic battery round the second finger, at a distance of three-quarters of an inch from the tip, and the other wire round the finger at the base. A gentle current was passed. For a brief period I was not conscious of the shocks, but suddenly the portion of finger included between the wires, from being white, became red and injected, and therewith there was excited a degree of pain that was unendurable. By removing the wires and applying cold once more, the acute pain passed off. But the most interesting point is, that while the first finger regained its normal sensibility in the course of an hour, and the second regained its normal sensibility in the parts which had been enclosed between the wires, the end of this second finger, from the point beyond which the upper wire had encircled it, remained completely insensible for four hours, and felt slightly numbed even thirty-six hours later.

From these experiments I have no alternative but to believe that the electric current cannot, according to our present knowledge of its application, be made practicable for the production of local anæsthesia.

The only way by which, as I would suggest, electric shocks can in any way be said to remove pain locally, is, that the pain which they excite creates a diversion, so that any new pain which may be inflicted on the part is not felt the less, but is lost in some degree in the pain which is preexistent. I give a simple illustration. The school-boy tells his new comrade that he can remove a hair from his head without the removal being felt. The skilful operator seizes a hair with his left thumb and finger, pulls it out quickly, and at the very moment strikes the head of his dupe a smart blow with his flat right hand.

The operation is performed, and it may be without the pain which would have been elicited by a simple pull. The pain, however, is not removed, but diverted. When my finger was painfully affected by the electric current, the entrance of the lancet or needle point into the skin caused sometimes a more acute, sometimes a less defined pain than is ordinary. Mr. Louis Parnell, who allowed me to perform some experiments on his finger, stated that his sensations were the same.

We have seen, nevertheless, in the first experiment related, that a powerful electric shock, sent through the whole body, will produce insensibility; why, therefore, should it not have the same effect in its local application? A dose of aconite tincture will render a body generally, insensible to pain; a drop of the same tincture, put on the lip, will produce numbness of the lip. Here is brought out at once a general and a local effect, each alike in kind, but different in degree. Why, then, should not the same obtain with the electric shock? To answer this, the consideration of the modes in which insensibility is ordinarily produced is necessary.

My late friend, Dr. John Snow, did much in clearing up the mystery which interposes here. He has described, and, to my mind, proved, that the sensibility of the body may be destroyed in two ways. First, it may be destroyed by the direct effect of some benumbing agent on the extremities of the nerves of the part. Secondly, by the effect of the agent on the centres of intelligence, *i. e.* by the destruction of consciousness. Thus, the effect of some narcotic vapors, taken into the system at large, may even be contrasted; amylene, for example, acts mainly on the extremities of the sensory nerves, interfering but feebly with the consciousness. Ether, on the other hand, suspends sensibility in proportion as it destroys consciousness.

Now, when a powerful electric shock is sent through an animal body it destroys sensibility, not because it is a local anæsthetic, but because it strikes out at a blow the consciousness. The animal, for the time, is dead to all impressions, pleasant or painful. An animal stunned by a blow is in the same condition; an animal in syncope is in the same condition. In this explanation I discover the reasons why electricity, as generally applied, *did*, and as locally applied, did *not*, produce insensibility.

In sending this paper to the press, it is no part of my object to interfere with the labors of Mr. Snape. The present unsatisfactory modes of preventing pain in surgical operations must unquestionably be superseded ultimately by some better process. It shall be read of some day as rude science, that leads the whole body into the realm of dissolution, that one poor molar may be dragged out without a flinch. Dr. Arnott thinks it so now. A mode of producing local anæsthesia, I mean complete anæsthesia, lies open this moment as the grandest practical discovery to be made in Medicine; and he who makes it can be begrudged his well-earned fame by none but the selfish and the foolish. I write, therefore, not critically, but to record my own researches and their results.

12, Hinde street, W. Sept. 4, 1858.

[*London Medical Times and Gazette.*

